# Is CFTR-delF508 Really Absent from the Apical Membrane of the Airway Epithelium?

**DOI:** 10.1371/journal.pone.0023226

**Published:** 2011-08-03

**Authors:** Lee A. Borthwick, Phil Botha, Bernard Verdon, Malcolm J. Brodlie, Aaron Gardner, David Bourn, Gail E. Johnson, Mike A. Gray, Andrew J. Fisher

**Affiliations:** 1 Institute of Cellular Medicine, Newcastle University, Newcastle upon Tyne, United Kingdom; 2 Institute for Cell & Molecular Biosciences, Newcastle University, Newcastle upon Tyne, United Kingdom; 3 Institute of Human Genetics, Newcastle University, Newcastle upon Tyne, United Kingdom; University of Minnesota, United States of America

## Abstract

**Background:**

Understanding where mutant CFTR is localised in airway epithelia is essential in guiding the best therapeutic approach to correct the dysfunction of the CFTR protein. The widely held paradigm is that CF patients harbouring the commonest mutation, CFTR-delF508, trap CFTR within the endoplasmic reticulum and target it for degradation. However there are conflicting reports concerning expression and localisation of CFTR-delF508 in lung tissue. To attempt to resolve this fundamental issue we developed a novel approach to measure CFTR-delF508 in the lower airways of patients who have undergone lung transplantation for advanced CF. By sampling CF and non-CF epithelium simultaneously from the same individual, confounding factors of different airway microenvironments which may have influenced previous observations can be overcome.

**Methods:**

Epithelia sampled by bronchial brushing above (CF) and below (non-CF) the bronchial anastomosis were stained for CFTR and the localisation and level of expression assessed (n = 12).

**Results:**

There was no significant difference in the proportion of tall columnar cells showing CFTR immunostaining as a discrete band at the apical membrane in cells harbouring the CFTR-delF508 mutation compared to non-CF cells (p = 0.21, n = 12). However, the amount of CFTR expressed at the apical surface was reduced by ∼50% in CF cells compared to non-CF cells (p = 0.04, n = 5).

**Conclusions:**

Our novel observation challenges the prevailing paradigm that CFTR is essentially absent from the apical membrane of respiratory cells harbouring the CFTR-delF508 mutation. Moreover, it raises the possibility that the new generation of CFTR potentiators may offer a realistic therapeutic option for CF patients.

## Introduction

Cystic Fibrosis (CF) is the most common autosomal recessive disease in Caucasians and the most common heritable cause of death during teenage and young adulthood [1]. CF is caused by mutations in the gene encoding the cystic fibrosis transmembrane conductance regulator (CFTR), a multidomain ATP-binding cassette protein responsible for the regulation of transmembrane transport of chloride and other ions. The most common mutation is a deletion of a phenylalanine residue at position 508 (CFTR-delF508), responsible for 70–80% of CF phenotype worldwide [2]. Current consensus is that this mutation leads to mislocalisation of CFTR from the apical membrane [3]. Absence of CFTR chloride secretion has been postulated to reduce airway surface liquid volume and impair mucocillary clearance and innate defence mechanisms [4]. These functional defects predispose the lungs to bacterial infection, inflammatory destruction and eventual death of the affected individual from respiratory failure.

The mislocalisation of CFTR has been observed in epithelial tissues from the lung [3], [5], [6], intestine [7] and sweat glands [8] under conditions of heterologous expression in culture, but also *in-vivo*. However, the sensitivity of CFTR to its microenvironment and the difficulties in accurately localising it has led to conflicting reports and suggestions that localisation might differ between tissues [7]. CFTR processing and localisation is also susceptible to alterations in temperature [9], [10], pH [11] and the presence of micro-organisms [12], [13]. Furthermore, the exact quantity of normal-functioning CFTR needed at the apical membrane for adequate function remains uncertain [14]. The correction of the mislocalisation of mutant CFTR has been a therapeutic goal for well over a decade and work continues to evaluate the role of gene therapy in correcting the underlying defects [15]. More recently there is increasing interest in the role of pharmacological agents which may affect CFTR localisation in respiratory epithelium [16], [17], [18], [19], [20]. To assess the efficacy of these approaches it is critical to accurately determine the localisation of CFTR in the epithelia of the lower airway as a marker of clinical phenotype and also to assess therapeutic interventions targeting CFTR.

To investigate CFTR localisation in the lower airway, we developed a novel approach of sampling the lower respiratory tract epithelia in CF patients who have undergone lung transplantation. This approach allows both non-CF cells and cells carrying the CFTR-delF508 mutation to be obtained from the same airway microenvironment simultaneously. This approach overcomes many of the significant limitations of previous studies, and may provide a possible testing ground for novel therapies.

## Materials and Methods

### Ethics statement

The Newcastle and North Tyneside Local Regional Ethics Committee approved the study (REC reference no 2001/179), and written informed consent was obtained from each subject.

### Characteristics of patients

Bronchial brushings were obtained using a protected specimen single-sheathed nylon cytology brush (5 fr; Wilson-Cook) from at least 2 cm above the airway anastomosis in the left main bronchus and at least 2 cm below the airway anastomosis in the segmental bronchus of the left lower lobe. Brushings were obtained from twelve (8F∶4M) lung transplant recipients between 79 and 357 days post bilateral lung transplantation for advanced CF lung disease. Standard immnuosuppression protocol after transplantation comprises a calcineurin inhibitor (ciclosporin/tacrolimus), a cell cycle inhibitor (azathioprine/mycophenolate mofetil) and oral corticosteroids (maintenance dose 0.1 mg/kg). All subjects showed no evidence of infection or acute or chronic rejection (grade A2 or above (ISHLT classification) and were bronchiolitis obliterans syndrome stage 0 (FEV1 >90% of baseline)).

### CF mutation analysis

See Supporting Information S1. Briefly, genomic DNA was extracted from cells using the Q-Card EZ1 DNA Tissue kit according to manufacturer's protocol (Qiagen). Genomic DNA was analysed for 28 CF mutations using the CF-HTv3 kit (Tepnel). Following amplification, separation and detection of products was performed on an ABI PRISM 3130*xl* Genetic Analyser and data analysed using Genemapper v3.7 (Applied Biosystems).

### Immunofluorescence

Cells isolated by bronchial brushing were smeared onto microscope slides and fixed with 4% paraformaldehyde. Cells were incubated with either MATG1061 (raised against amino acids 503–515 in the N-terminal) (RD-Biotech), 570 (raised against amino acids 731–742 in the R-domain) or 596 (raised against amino acids 1204–1211 in nucleotide binding domain 2) (both Cystic Fibrosis Foundation) anti-CFTR monoclonal antibodies and anti-Interferon regulatory factor-1 (IRF-1 - Santa Cruz) or anti-β-tubulin polyclonal antibodies (Sigma). Antigen-antibody complexes were detected using appropriate flourochrome-linked secondary antibodies with DAPI as a nuclear counterstain. Laser settings for each patient were optimised using the non-CF cells and the same settings used to compare CFTR expression in the CFTR-delF508 cells. Images acquired using a Leica TCS-SP-2UV laser scanning confocal microscope. Isotype matched immunoglobulins were employed as negative controls.

### Statistical Analysis

The percentage of tall columnar epithelial (TCE) cells expressing CFTR as a distinct apical band was examined in multiple randomly selected fields and compared above and below the airway anastomosis from each individual. Patients with <100 cells were excluded. Results were validated by counts from two blinded individuals. Total and average pixel intensity of CFTR staining was quantified using Photoshop CS3 (Adobe) in multiple randomly selected fields and compared above and below the airway anastomosis from each individual. At least 20 cells/sample were assessed. The difference between groups was assessed by a one way ANOVA using SPSS 14.0. Differences with a p-value of <0.05 were considered statistically significant.

### Human Lung Tissue Sampling

Normal control tissue was obtained from unused donor lungs. Lung samples were fixed in 10% formalin, embedded, sectioned and stained with antibodies against E-cadherin (BD Bioscience) and CFTR (RD-Biotech). Antibodies were detected using appropriate flourochrome-linked secondary antibodies with DAPI as a nuclear counterstain. Areas of intact epithelium were identified and images acquired using a LSM 510 laser scanning confocal microscope. Isotype matched immunoglobulins were employed as negative controls.

## Results

**Figure 1:**
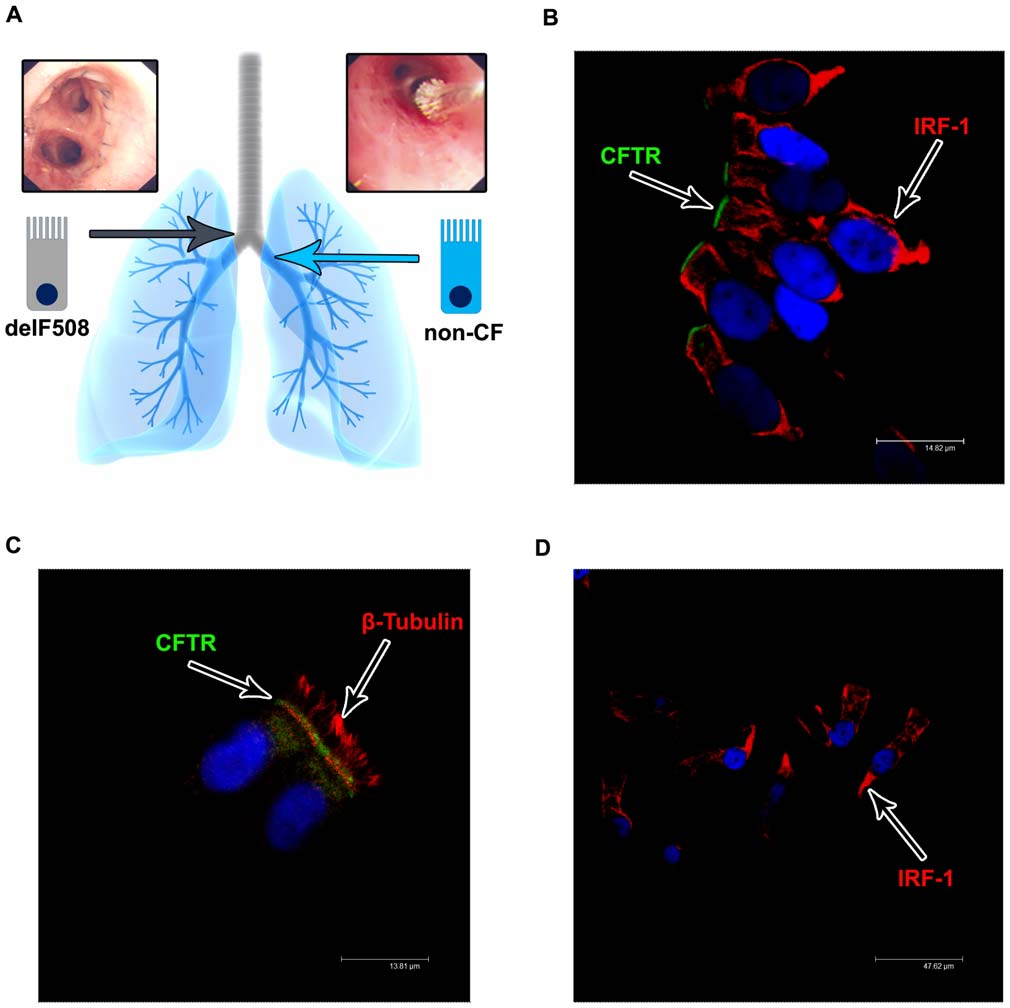
The post transplant lung in a CF patient; a novel way to investigate CFTR localisation. A) Bronchial brushings (example shown in the upper right panel) from post transplant CF patients allowed both CFTR-delF508 affected epithelial cells (above the airway anastomosis from the native tissue) and non-CF epithelial cells (below the airway anastomosis from the donor lung) to be obtained from the same patient and investigate CFTR localisation and expression levels. B+C) Bronchial brushings of non-CF cells fixed in 4% paraformaldehyde and stained for CFTR (MATG1061 - FITC/Green) and IRF-1 (B) or β-tubulin (C) (TRITC/Red) with a nuclear counter stain (DAPI/Blue). CFTR is localised predominantly to the apical membrane of tall columnar epithelial cells. Images acquired on a Leica confocal microscope (×100 magnification). D) Corresponding isotype control. CFTR (MATG1061) was replaced by IgG_2a_ at the corresponding concentrations. No non-specific staining was seen in tall columnar epithelial cells. Images acquired on a Leica confocal microscope (×63 magnification).

**Figure 2:**
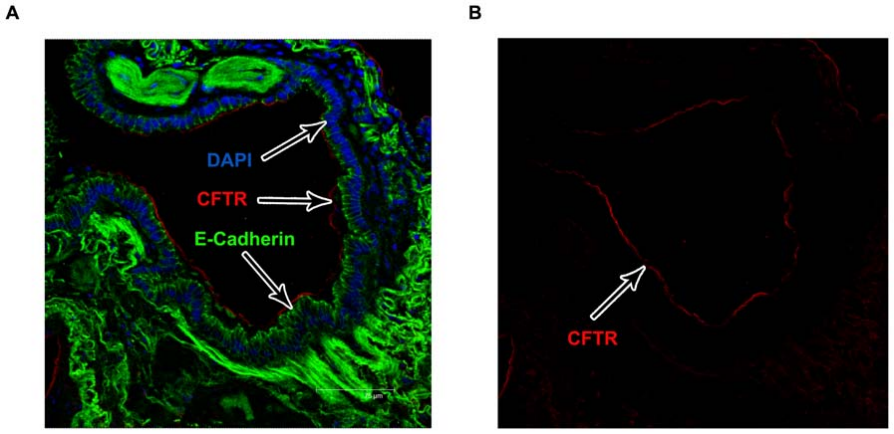
CFTR expression in explanted lung tissue. A) Explanted non-CF lung tissue was stained for CFTR (MATG1061 - TRITC/Red) and the epithelial marker E-cadherin (FITC/Green) with a nuclear counter stain (DAPI/Blue). CFTR is expressed only at the apical membrane of airway epithelial cells. Images acquired on a Leica confocal microscope (×40 magnification). B) Image acquired in A) showing CFTR (TRITC/Red) staining only.

To investigate CFTR expression in both non-CF and CF cells from the lower airway in the same individual, epithelia were sampled by bronchial brushing from above (CFTR-delF508 mutation) and below (non-CF) the airway anastomosis joining the native bronchus and the transplanted lung (Fig. 1A). Cells from above the anastomosis were confirmed to be 100% homozygous for the CFTR-delF508 mutation by genetic analysis. CFTR protein expression and distribution was investigated using a well validated anti-CFTR monoclonal antibody, MATG1061. To aid identification and counting we co-stained the cytoplasm of the cells with IRF-1, a transcription factor that is localised throughout the cytoplasm of epithelial cells, as well as DAPI to stain the nucleus. Consistent with previous results using the MATG1061 antibody in freshly isolated nasal and bronchial epithelia cells [3], [5], [21] and freshly excised human tissue [7], [22] we found that the majority of non-CF cells showing a strong CFTR signal were TCE cells and CFTR appeared as a discrete band at the apical pole (Fig. 1B). Further analysis showed that the apical CFTR band was located just below the cilia (Fig. 1C) and that staining was absent in isotype matched immunoglobulins negative controls (Fig. 1D). A similar localisation was observed in the superficial epithelium of explanted lung tissue from a non-CF patient (red staining in Fig. 2).

**Figure 3:**
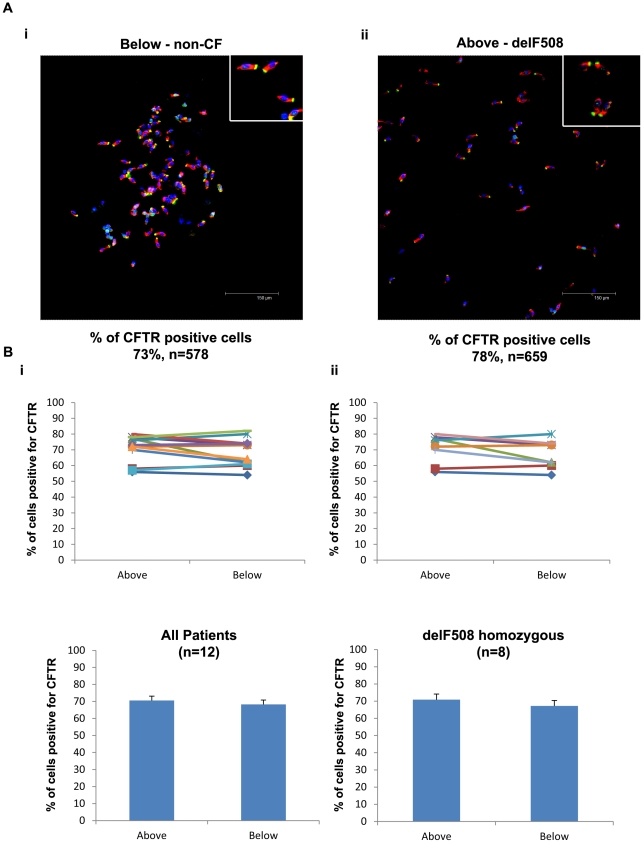
CFTR is expressed apically in CFTR-delF508 affected epithelial cells. A) Example of bronchial brushings of non-CF (i) and CFTR-delF508 (ii) affected epithelial cells fixed in 4% paraformaldehyde and stained for CFTR (MATG1061 - FITC/Green) and IRF-1 (TRITC/Red) with a nuclear counter stain (DAPI/Blue). Quantification displayed refers to the specific patient the images were acquired from. Images acquired on a Leica confocal microscope (×20 magnification). B) Quantification of the percentage of tall columnar epithelial cells positive for CFTR at the apical membrane. i) All patients (CFTR-delF508 homozygous patients (N = 8) and CFTR-delF508 heterozygous patients (N = 4)). No significant difference in the percentage of cells expressing CFTR at the apical membrane was seen in non-CF (68±3%) and CF (71±3%) epithelial cells (p = 0.21, N = 12). ii) CFTR-delF508 homozygous patients only (N = 8). No significant difference in the percentage of cells expressing CFTR at the apical membrane was seen in non-CF (67±3%) and CFTR-delF508 homozygous affected (71±3%) epithelial cells (p = 0.14, N = 8). There was also no significant difference in the total number of cells quantified (p = 0.48, N = 8).

To quantify the number of non-CF and CF TCE cells expressing CFTR at the apical pole we collected data from at least 100 cells/sample, and the number of CFTR positive cells was quantified by two blinded observers. An example of a typical image is shown in figure 3A. These images show a group of ∼40 cells and it can clearly be seen that the majority of TCE cells express a clear apical band for CFTR in both samples. Mean data from twelve lung recipient patients with CF collected in an identical way is presented in figure 3B. Results show that there was no significant difference in the percentage of TCE cells expressing apical CFTR above and below the airway anastomosis (Fig. 3Bi). Eight of the twelve lung transplant recipients were CFTR-delF508 homozygous and four were CFTR-delF508 heterozygous. In all cases for the individuals heterozygous for the CFTR-delF508 mutation the second mutant allele differed making it difficult to analyse the data as one group as it would be very difficult to interpret any findings with confidence due to the possible effects of the different 2^nd^ mutations on CFTR localisation/expression levels. Furthermore, due to the limited number of CF patients who undergo transplantation it would be extremely difficult to generate sufficient data sets to achieve significance using any individual second mutation.

Consequently we analysed the results from CFTR-delF508 homozygous patients alone. Again, there was no significant difference in the percentage of TCE cells expressing apical CFTR above and below the airway anastomosis (Fig. 3bii). These results clearly indicate that the proportion of cells expressing a distinct band of apical CFTR is not different between non-CF and CFTR-delF508 CF cells.

To confirm the results above with the MATG1061 antibody we proceeded to compare the number of non-CF and CF TCE cells expressing CFTR at the apical pole with 2 further well characterised [23], [24] CFTR monoclonal antibodies (570 and 596) in a single patient (Fig. S1). We found that the percentage of TCE cells expressing CFTR at the apical pole above and below the airway anastomosis was very similar using all 3 antibodies (MATG1061: 74% vs 72%, 570: 70% vs 66%, 596: 64% vs 67%) confirming the accuracy of the MATG1061 antibody.

**Figure 4:**
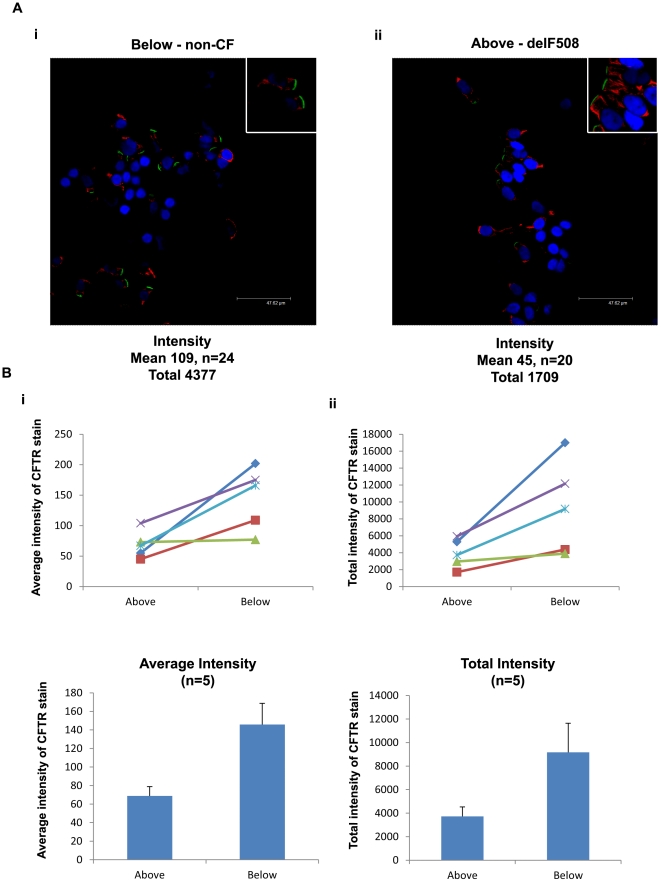
Reduction in the expression of CFTR-delF508 CFTR at the apical membrane. A) Example of bronchial brushings of non-CF (i) and CFTR-delF508 (ii) affected epithelial cells fixed in 4% paraformaldehyde and stained for CFTR (MATG1061 - FITC/Green) and IRF-1 (TRITC/Red) with a nuclear counter stain (DAPI/Blue). Quantification displayed refers to the specific patient the images were acquired from. Images acquired on a Leica confocal microscope (×63 magnification). B) Quantification of the average (i) and total (ii) pixel intensity of CFTR staining at the apical membrane of tall columnar epithelial cells (N = 5). There was a significant reduction in the average pixel intensity (above 69±10 vs below 146±23, p = 0.03, n = 5) and the total pixel intensity (above 3729±805 vs below 9173±2465, p = 0.04, n = 5) of CFTR staining at the apical membrane in CFTR-delF508 affected epithelial cells compared to non-CF. There was no significant difference in the total number of cells counted (p = 0.31, N = 5), or the total number of pixels quantified (p = 0.17, N = 5).

The finding that CFTR was detected in the same proportion of non-CF and CF lower airway cells was unexpected since impaired trafficking of CFTR-delF508 is thought to be the hallmark of CF in airway epithelial cells [3]. However, the CFTR-delF508 mutation has also been shown to reduce protein stability at the cell surface [23] and may therefore decrease the quantity of mutant protein at the apical membrane. To investigate this possibility we used image analysis software to quantify the signal intensity of the apical band of CFTR. We analysed at least 20 cells/sample from non-CF (Fig. 4Ai) and CF (Fig. 4Aii) cells and calculated the average and total pixel intensity for each isolated apical band (Fig. 4B). In contrast to the data on the proportion of CFTR positive cells, we found a significant reduction in both the average (Fig. 4Bi) and total (Fig. 4Bii) pixel intensity of CFTR staining at the apical membrane of CF cells compared to non-CF cells. These results, together with the data in figure 3, strongly suggest that the CFTR-delF508 protein in lower airway TCE cells does not exhibit a major trafficking defect *per se*, but the mutation does markedly reduce the quantity of protein localised to the membrane.

## Discussion

The rationale for developing gene therapy and small molecule drug corrector approaches to treat CF lung disease is the reported absence of CFTR from the apical membrane of airway epithelium in the commonest mutation, CFTR-delF508. This is believed to occur due to inefficient biosynthetic maturation of the mutant protein and its rapid degradation *via* the endoplasmic reticulum-associated degradation pathway (ERAD) [25], [26]. This paradigm is based historically on *in vitro* studies in cells expressing exogenous mutant CFTR as well as CFTR localisation studies in both fixed excised lung tissue and cultured nasal epithelium [3], [5], [6], [27]. These studies, however, have significant limitations due to overexpression and processing artefacts and inherent microenvironmental differences between the CF and non-CF airway which may influence CFTR localisation and expression. Indeed recent studies have shown that endogenous CFTR is processed and trafficked differently to exogenous CFTR [28] and that processing and function of CFTR is species dependant [29]. Typically, only nasal epithelium is sampled *in vivo* in CF patients, and uncertainty exists as to the relation of CFTR expression in the upper and lower airway. We therefore developed a novel approach which overcomes these problems to assess CFTR localisation in lower airway epithelial cells from patients who have undergone lung transplantation for advanced CF.

Cells brushed from above the bronchial anastomosis in CFTR-delF508 homozygous individuals were confirmed to be 100% CFTR-delF508 homozygous and those from below were confirmed non-CF. We found no significant difference in the percentage of TCE cells expressing apical CFTR when sampled from below (non-CF) and above (CFTR-delF508) the airway anastomosis. However, apical CFTR staining showed a significant reduction in both average and total pixel intensity in CFTR-delF508 affected cells compared to non-CF cells. This suggests that, when TCE cells are isolated from the same airway microenvironment, an equal proportion of CFTR-delF508 and non-CF cells express CFTR at the apical membrane. In contrast to some previous reports, the quantity of CFTR expressed at the apical membrane of TCE cells was the only distinguishing feature in the lower airway.

There have been conflicting reports regarding CFTR expression in excised native airway tissues. Using the same antibody as in the present study, frozen sections of excised nasal polyps were shown to express CFTR predominantly in the apical membrane and sub-membranous compartment of ciliated epithelial cells. This pattern of expression was also seen in CFTR-delF508 homozygous tissues, but with additional accumulation along the basolateral and mitochondrial membranes [6]. This antibody was raised against a synthetic peptide corresponding to amino acid position 503–515 and has been repeatedly shown to identify normal and CFTR-delF508 equally [3], [5], [6]. In keeping with our findings, Kalin *et al* also found indistinguishable levels of CFTR expression in pseudostratified columnar epithelium covering nasal polyps, and in submucosal glands of nasal polyps [7]. This study included cellular localisation and signal intensity analysed by immunohistochemistry. Only submucosal gland CFTR expression was analysed in bronchial tissue and was found to be indistinguishable in CF and non-CF individuals. The same study found CFTR expression undetectable in bronchial specimens using the MATG1061 antibody. The specificity of this antibody has also been questioned, after non-specific staining was demonstrated in sweat glands [8]. It has, however, been pointed out that the anti-alkaline phosphatase method used in preference of immunofluorescence by these authors may have resulted in a decrease in specificity [5]. Antibodies raised to other domains of CFTR have delivered contrasting results. Using an antibody raised to a C-terminal peptide (residues 1468–1480) Engelhardt *et al* found no evidence of CFTR protein expression in the bronchial surface epithelium, apart from localised expression in a small number of flask-like cells [30]. The expression of CFTR was limited to the submucosal glands in frozen bronchial tissue specimens, and completely absent in CFTR-delF508 homozygous individuals. In contrast, using antibodies raised against non-overlapping regions of NBD2, Kreda *et al* found no evidence of expression of CFTR-delF508 CFTR in TCE cells of human bronchial cryosections obtained from homozygous individuals despite detecting CFTR expression in the same cells from non-CF tissue [24].

Studies utilising detached airway epithelial cells, in particular nasal epithelial cells, have generally demonstrated identifiable differences in CFTR expression between CFTR-delF508 homozygous and non-CF individuals. Penque *et al.* found CFTR to be localised mainly in the apical region of ciliated and non-ciliated TCE cells [3]. Using 3 different antibodies, including MATG1061, apically localised CFTR was identified in 22% of CFTR-delF508 homozygous individuals, 42% of heterozygotes and 56% in healthy individuals. Carvalho-Oliveira *et al.* confirmed these findings using a panel of 7 anti-CFTR antibodies and a range of processing and fixation techniques [5]. Dormer *et al* found a distinct peri-nuclear staining in TCE cells obtained by nasal brushing from CFTR-delF508 homozygous individuals, and similar proportional expression of apical CFTR from CF/non-CF individuals [27]. The former finding could not be confirmed by Carvalho-Oliveira *et al.* using the same antibody and fixation technique [5]. Our analysis of CFTR expression in TCE cells from the lower airway therefore suggests a marked difference in localisation between the upper and lower airway, when taken together with these previous reports.

A possible alternative explanation for our findings is that the particular microenvironment in the transplanted lung may correct the CFTR-delF508 trafficking defect. Pharmacological therapy is common in the lung transplant recipient, particularly the use of various immunosuppressants, and this may affect CFTR maturation and trafficking. Ciclosporin has been demonstrated to correct processing mutations of other ATP-binding cassette proteins, particularly P-glycoprotein, but failed to demonstrate any such effect in cell lines expressing CFTR-delF508 [31], [32]. However, no direct evidence of an increased expression in response to any of the other commonly used immunosuppressive agents exists.

Our novel observation challenges the prevailing paradigm that CFTR is essentially absent from the apical membrane of respiratory cells in CF patients harbouring the CFTR-delF508 mutation. Instead these data suggest that CFTR is widely expressed at the apical membrane in epithelial cells from the lower airway but at a significantly reduced level compared to non-CF cells. This raises the exciting possibility that the new generation of drugs which potentiate mutant CFTR activity [33], [34], [35] may offer a realistic therapeutic option for CF patients, obviating the need for gene therapy.

## Supporting Information

Figure S1 **Comparison of CFTR localisation using 3 different CFTR antibodies.** Bronchial brushings of non-CF and CF cells were stained for CFTR (left panel: MATG1061, centre panel: 570, right panel: 596, (Cystic Fibrosis Foundation (CFF)) - FITC/Green) and IRF-1 (TRITC/Red) with a nuclear counter stain (DAPI/Blue). CFTR is localised predominantly to the apical membrane of tall columnar epithelial cells in both non-CF and CF cells for all 3 antibodies tested. Images acquired on a Leica confocal microscope (×20 magnification).
(TIF)

Supporting Information S1 **Supplementary Material and Methods.**
(DOCX)
