# Supplementary figures and images for: Is CFTR-delF508 Really Absent from the Apical Membrane of the Airway Epithelium?
Source: PLoS One. 2011 Aug 3;6(8):e23226. doi: 10.1371/journal.pone.0023226 (PMC3149652; doi:10.1371/journal.pone.0023226)

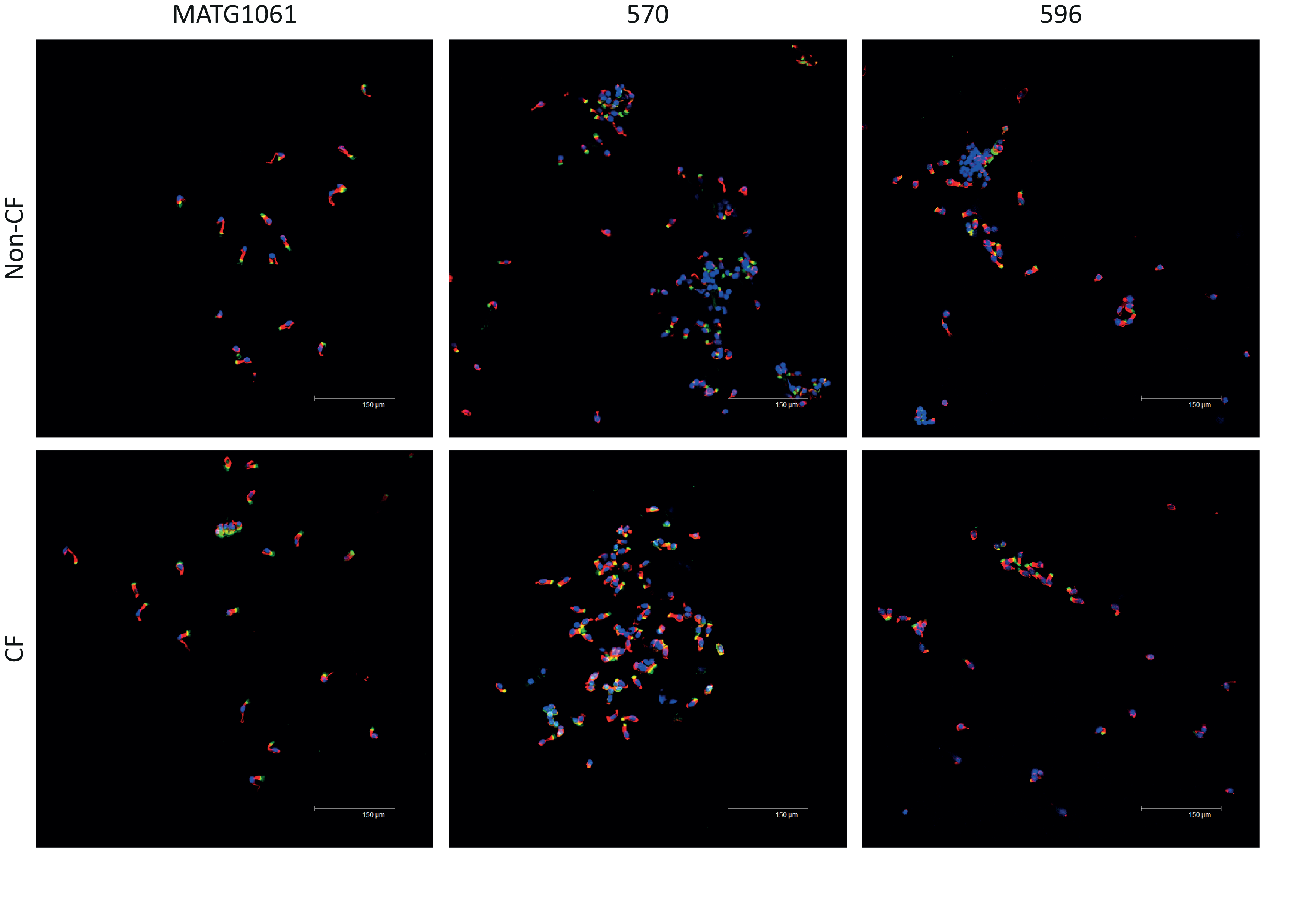

Supplement: Figure S1 — Comparison of CFTR localisation using 3 different CFTR antibodies. Bronchial brushings of non-CF and CF cells were stained for CFTR (left panel: MATG1061, centre panel: 570, right panel: 596, (Cystic Fibrosis Foundation (CFF)) - FITC/Green) and IRF-1 (TRITC/Red) with a nuclear counter stain (DAPI/Blue). CFTR is localised predominantly to the apical membrane of tall columnar epithelial cells in both non-CF and CF cells for all 3 antibodies tested. Images acquired on a Leica confocal microscope (×20 magnification). (TIF) [file pone.0023226.s001.tif]
