# Supplementary material for: Is CFTR-delF508 Really Absent from the Apical Membrane of the Airway Epithelium?
Source: PLoS One. 2011 Aug 3;6(8):e23226. doi: 10.1371/journal.pone.0023226 (PMC3149652; doi:10.1371/journal.pone.0023226)
Supplement: Supporting Information S1 — Supplementary Material and Methods. (DOCX) [file pone.0023226.s002.docx]

**Is CFTR-delF508 really absent from the apical membrane of the airway epithelium?**

Lee A Borthwick, Phil Botha, Bernard Verdon, Malcolm Brodlie, Aaron Gardner, David Bourn, Gail E Johnson, Mike A Gray, Andrew J Fisher.

**Supplementary Material and Methods**

**Genomic DNA extraction**

Bronchial epithelial cell extracts were prepared from brushings taken from transplant patients homozygous for the CFTR-delF508. Cells were pelleted and then resuspended in 190 μl of buffer G2 (Qiagen). Following resuspension, 10 μl of proteinase K (Qiagen) was added and the samples were incubated overnight at 56ºC. Genomic DNA was extracted using the Q-Card EZ1 DNA Tissue kit and EZ1 robot following the manufacturer’s protocol (Qiagen).

**Cystic Fibrosis analysis in bronchial epithelial cells**

Following quantification using a spectrophotometer (Nanodrop), Genomic DNA was analysed for 28 cystic fibrosis mutations using the CF-HTv3 kit (Tepnel). PCR reactions were carried out in two mixes each in a total volume of 15μl containing 15 ng of genomic DNA, 1 X HSB buffer, 2.5 units of HS *Taq* DNA polymerase and 9 μl of primer set A or B (Tepnel). PCR amplification was performed in a M J Research tetrad peltier thermal cycler at 94ºC for 20 mins, followed by 30 cycles of 94ºC for 1 min, 58ºC for 2 mins and 72ºC for 1 min, followed by a final extension at 72ºC for 20 mins.

Following amplification, separation and detection of products was performed on an ABI PRISM 3130*xl* Genetic Analyser (Applied Biosystems). Two microlitres of the PCR product were diluted in 23.2 μl of water and 0.8 μl of GeneScan^®^ 400HD (Rox) size standard (Applied Biosystems), denatured at 94ºC for 3 mins and cooled on ice. Samples were injected for 3 secs at 15kV then electrophoresed for 600 secs at 15kV using Performance Optimised Polymer (POP) 7 and a 36 cm length-to-detector uncoated capillary. Data was analysed using Genemapper v3.7 software (Applied Biosystems).

**Testing for chimaerism in bronchial epithelial cell**

Genomic DNA was analysed for 16 polymorphic markers using the PowerPlex^®^ 16 System (Promega). PCR reactions were carried out in a volume of 12.5 μl containing 10ng of genomic DNA, 1X Gold Star buffer, 1X PowerPlex^®^ 16 primer pair mix and 2 units of AmpliTaq Gold DNA polymerase (Applied Biosystems). Amplification was performed in a GeneAmp PCR system 9700 thermal cycler (Applied Biosystems) at 95ºC for 11 mins, followed by 96ºC for 1 min, followed by 10 cycles of ramp 100% to 94ºC for 30 s, ramp 29% to 60ºC for 30 s and ramp 23% to 70ºC for 45 s, then 22 cycles of ramp 100% to 90ºC for 30 s, ramp 29% to 60ºC for 30 s and ramp 23% to 70ºC for 45 s, followed by a final extension at 60ºC for 30 mins. One microlitre of PCR product was diluted in 14 μl of water and 1 μl of ILS600 size standard (Promega), denatured at 94ºC for 3 mins and cooled on ice. The same treatment was carried out with 1 μl of PowerPlex^®^ 16 System allelic ladder (Promega) and samples were injected for 3 s at 15kV then electrophoresed for 20 mins at 15kV using POP 7 and a 36cm length-to-detector uncoated capillary on a 3130*xl* Genetic Analyser (Applied Biosystems). Data was analysed using GeneMarker v1.85 software (Soft Genetics). The peak area of each allele is measured and the percentage of donor cells present in the sample is calculated using the following formula

| peak area of each donor allele present | = % donor contribution |
| --- | --- |
| peak area of each donor allele + peak area of each recipient allele present |  |
